# Comparative evaluation of the web-based contiguous cartogram generation tool go-cart.io

**DOI:** 10.1371/journal.pone.0298192

**Published:** 2024-05-08

**Authors:** Ian K. Duncan, Michael T. Gastner

**Affiliations:** 1 Yale-NUS College, Singapore, Singapore; 2 Infocomm Technology, Singapore Institute of Technology, Singapore, Singapore; CCET: Chandigarh College of Engineering and Technology, INDIA

## Abstract

Area cartograms are map-based data visualizations in which the area of each map region is proportional to the data value it represents. Long utilized in print media, area cartograms have also become increasingly popular online, often accompanying news articles and blog posts. Despite their popularity, there is a dearth of cartogram generation tools accessible to non-technical users unfamiliar with Geographic Information Systems software. Few tools support the generation of contiguous cartograms (i.e., area cartograms that faithfully represent the spatial adjacency of neighboring regions). We thus reviewed existing contiguous cartogram software and compared two web-based cartogram tools: fBlog and go-cart.io. We experimentally evaluated their usability through a user study comprising cartogram generation and analysis tasks. The System Usability Scale was adopted to quantify how participants perceived the usability of both tools. We also collected written feedback from participants to determine the main challenges faced while using the software. Participants generally rated go-cart.io as being more usable than fBlog. Compared to fBlog, go-cart.io offers a greater variety of built-in maps and allows importing data values by file upload. Still, our results suggest that even go-cart.io suffers from poor usability because the graphical user interface is complex and data can only be imported as a comma-separated-values file. We also propose changes to go-cart.io and make general recommendations for web-based cartogram tools to address these concerns.

## Introduction

**Fig 1:**
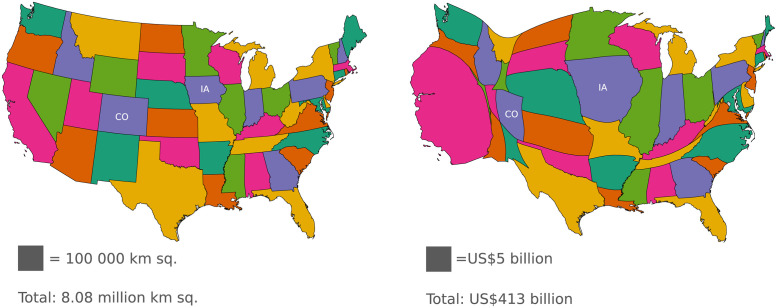
Example cartogram. Conventional map (left) and contiguous cartogram (right) of 2018 agriculture sector output by state in the United States [1]. Colorado (CO) and Iowa (IA) are labeled in both maps.

Area cartograms are map-based data visualizations in which the area of each map region (e.g., state or province) is proportional to some numeric data value (e.g., population or gross domestic product). An area cartogram is considered to be contiguous if it preserves conventional map topology such that neighboring regions on a conventional (e.g., equal-area) map remain neighbors in the cartogram, and vice versa. Consider the contiguous cartogram in Fig 1 displaying agriculture sector output by state in the United States in 2018. Notice that, while Colorado (CO) appears larger in area than Iowa (IA) in the conventional map because Colorado has more land area, Iowa’s area appears over three times larger than that of Colorado in the cartogram. The proportion of the cartogram areas reflects that Iowa’s agriculture sector output of US$29.5 billion is more than three times higher than Colorado’s agriculture sector output of US$8.1 billion [1]. While various types of non-contiguous cartograms exist, we focus on contiguous cartograms, which performed well in a previous evaluation that compared different cartogram types [2].

First becoming popular in print media in the early 20th century [3, 4], widespread adoption of computer technology and the Internet over the last three decades has created new opportunities for cartograms to be presented electronically [5]. Consequently, cartograms have become popular in online media as an accompaniment to news articles, especially those that display statistics, such as election results [6–8].

Despite their popularity, there is a dearth of software tools for generating cartograms that are accessible to non-technical users unfamiliar with traditional Geographic Information Systems (GIS) software. A survey of cartogram generation tools by Markowska and Korycka-Skorupa [9] revealed several web and desktop applications. However, presently, many of these tools are either no longer functional or are designed to be used in conjunction with a GIS software package, rendering them firmly out of reach of non-technical users. Markowska and Korycka-Skorupa [9] do not mention the fBlog Online Cartogram Tool, which provides a simple web interface for users wanting to generate cartograms of the United States and Europe [10]. While its feature set is limited, it remains one of the only such easy-to-use software tools that is in working order. After the survey by Markowska and Korycka-Skorupa [9], Viry et al. [11] developed the online mapping application Magrit, which generates cartograms from preinstalled sample basemaps and provides an attractive graphical user interface. However, its documentation is only available in French, which was not as widely understood as English in the participant pool available for this study.

To address this lack of an easy-to-use and effective cartogram generation software, Shi, Duncan, and Gastner [12] developed go-cart.io, a web application aimed at non-technical users that generates cartograms of a variety of geographies from uploaded data. Herein, we present the results of an experiment we carried out to evaluate the usability of go-cart.io and fBlog, which were, to the best of our knowledge, the only two functional web-based cartogram generation tools available with user manuals in English at the time of the experiment. Participants were required to generate a cartogram of a provided data set with go-cart.io and fBlog. The order in which participants encountered the two cartogram tools was randomized. Afterwards, participants referred to a figure generated from their cartogram to answer questions about the data set. Finally, participants rated the usability of both cartogram generation tools based on the widely adopted System Usability Scale and left additional written feedback. Based on the experimental results, we make recommendations for the design of future versions of web-based cartogram generation tools.

## Related work

### Usability of GIS software

The International Organization for Standardization [13] defines system usability as the combination of three criteria: effectiveness, efficiency, and satisfaction. Komarkova, Jakoubek, and Hub [14] adopted these three criteria as their framework to evaluate GIS usability. Despite the increasing prevalence of geospatial data, Komarkova et al. [14] note that traditional GIS software tools suffer from poor usability. They are often desktop applications, requiring users to install and configure them before use. Often not designed with usability in mind [15], GIS user interfaces are usually complicated and require extensive training before they can be used. The reliance of certain GIS tools on proprietary file formats is also a concern for users who need integration with other software systems [14]. The emergence of web-based GIS tools presents an opportunity to address some of these concerns because these tools do not need to be installed and configured. However, web-based applications must overcome certain additional challenges. While it may be acceptable for desktop GIS packages to require extensive training for users to take advantage of their rich feature set, web applications must cater to users who are not tech-savvy and are often in a hurry.

In a survey of GIS usability studies, Unrau and Kray [15] pinpointed additional areas where GIS tools possess poor usability. Over half the tools surveyed were reported to give poor error messages that did not provide any indication of how to fix the problem encountered. Moreover, Unrau and Kray [15] found that many users failed to complete study tasks because the interface of the studied GIS tool provided no visual indication of how to proceed. These findings echo the results of a survey of users of desktop GIS software conducted by Davies and Medyckyj-Scott [16]. Participants in this survey also complained of “total nonsense” error messages and being “unsure what to do when they sit in front of a GIS” [16]. This latter point may result from the failure of many GIS software tools to conform to traditional norms of software interface layout, preventing users from applying general software skills to GIS systems. Davies and Medyckyj-Scott [16] also found that most GIS systems do not do enough to support novice and infrequent users. They suggested that GIS software should provide better documentation and online help because the presence of these features was associated with a higher usability rating by survey participants.

### Cartogram generation tools

Existing cartogram generation tools also suffer from some of the usability challenges discussed above. Markowska and Korycka-Skorupa [9] conducted an extensive survey of cartogram generation tools. Out of the five studied tools, three supported generating contiguous cartograms. One of these tools, MAPresso, relies on obsolete Java applet technology and is no longer usable [17]. Another tool, ScapeToad was a standalone desktop application but is no longer available online [18]. The third tool, a cartogram utility for ArcGIS [19], remains working but is inaccessible to non-technical users because it is a plugin for ArcGIS, a commercial GIS software package requiring a software license and specialized training to use effectively [9]. While all the above-mentioned tools aim for a high degree of automation, the recently developed Windows application Cartogram Studio [20] focuses on manual construction of cartograms. On the one hand, this feature makes Cartogram Studio an excellent didactic tool. On the other hand, manual customization of cartograms is time-consuming [21], limiting the practical usability of Cartogram Studio outside an educational setting.

Markowska and Korycka-Skorupa [9] quantify the performance of the generation tools they studied using a numeric scale that awards points for possessing certain functionality (e.g., whether the tool supports saving generated cartograms in a vector image format). However, they do not explicitly consider usability in their evaluation. This omission, combined with the development of new tools such as go-cart.io, provides an opportunity for an updated, usability-focused assessment of currently available cartogram generation tools.

### System Usability Scale

Brooke’s [22] 1996 System Usability Scale (SUS) questionnaire provides a standardized method of quantifying the usability of software and other systems. The questionnaire comprises ten statements alternating between positive and negative sentiment. Table 1 lists all the SUS items. The alternating sentiment of the questions ensures that participants carefully consider each questionnaire item [16]. Respondents indicate their agreement or disagreement with each statement on a 5-point Likert scale. Since its development, the SUS has become widely adopted and has been evaluated as one of the best performing surveys at measuring system usability [23].

**Table 1:** The 10 items of the System Usabilty Scale [22]. For each item, participants were asked to indicate their agreement with the item on a 5-point Likert scale.

№	Item Statement
1.	I think that I would like to use this system frequently.
2.	I found the system unnecessarily complex.
3.	I thought the system was easy to use.
4.	I think that I would need the support of a technical person to be able to use this system.
5.	I found the various functions in this system were well integrated.
6.	I thought there was too much inconsistency in this system.
7.	I would imagine that most people would learn to use this system very quickly.
8.	I found the system very cumbersome to use.
9.	I felt very confident using the system.
10.	I needed to learn a lot of things before I could get going with this system.

Unrau and Kray [15] also pointed out that the SUS has been adopted by many studies that evaluate GIS software specifically. Therefore, we adopted the SUS as the main measure of cartogram generation tool usability in our experiment.

## Overview of go-cart.io

**Fig 2:**
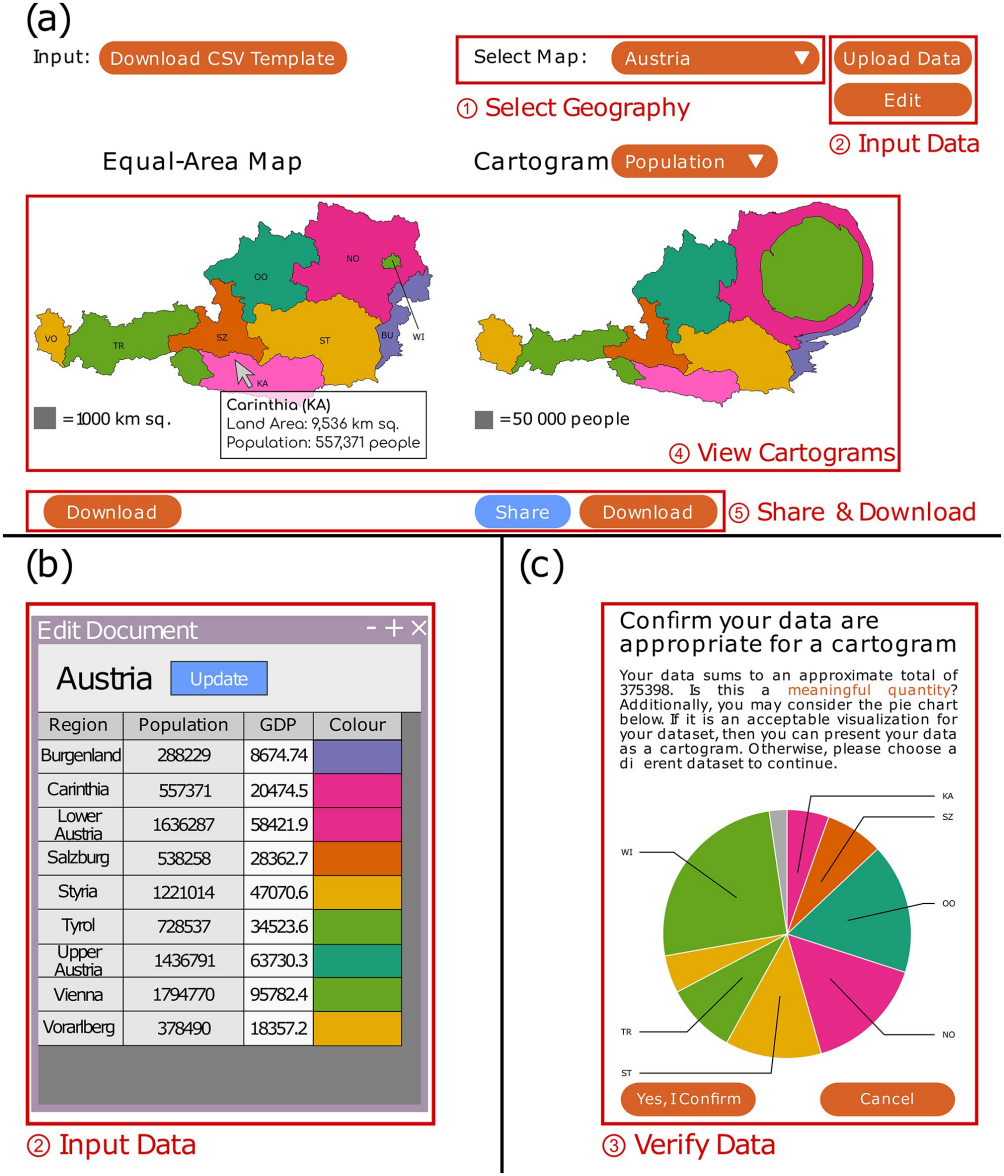
Screenshot of go-cart.io user interface. The main interface is presented in panel (a), the pop-up numeric data-entry interface in panel (b), and the pie-chart data verification interface in panel (c). Numbers in red circles indicate the sequence of steps required to create a cartogram (see main text for details).

The web application go-cart.io allows users to generate cartograms for a set of selected geographies using their own data. The generation tool uses modern web technologies, and runs in any up-to-date web browser without requiring installation of any additional software. Table 2 summarizes the features offered by go-cart.io as compared to existing cartogram generation tools. Cartograms are generated on a cloud server using the fast flow-based method developed by Gastner, Seguy, and More [24]. Fig 2 provides a screenshot of the go-cart.io interface. Numbers in the figure highlighting the user interface elements of go-cart.io correspond to the steps of the instructions below. To generate a cartogram, users must:

1. Select the geography for which they want to generate a cartogram from the drop-down list at the top-right. There are currently 82 available geographies on go-cart.io, including countries, sub-country divisions (e.g., states and provinces), and multinational political entities such as the European Union and ASEAN. A list of preinstalled maps is available in the online supplemental material on the publisher’s website.
2. Input numeric data and colors for each region of the selected geography. Users may do this in one of two ways:
  - Downloading a template spreadsheet in comma-separated-values (CSV) format by clicking the “Download CSV Template” button on the top-left. After entering numeric data as well as colors in hex-code format for each region, users may upload their edited CSV file by clicking the “Upload Data” button on the top-right.
  - Clicking the “Edit” button at the top-right and then entering the numeric data and color for each region in an editing interface that appears in a pop-up window, as shown in panel (b) of Fig 2.
3. Confirm that their numeric data are appropriate for a cartogram. In most cases, cartograms should only be used for data that add up to an interpretable total (e.g., absolute population or gross domestic product by region, but not gross domestic product per capita [25]). To aid users, go-cart.io displays the users’ numeric data in a pie chart, as shown in panel (c) of Fig 2. If the pie chart is an acceptable visualization of the users’ numeric data, they may proceed to generate the cartogram. Otherwise, they should select different numeric data.
4. Once go-cart.io generates a cartogram from the given data, users may preview and interact with it. Following recommendations from Dent [26], go-cart.io always displays generated cartograms alongside the corresponding conventional map and a square-shaped area-to-value legend as anchor stimulus, as shown in panel (a) of Fig 2. go-cart.io also provides the following interactive features:
  - **Infotip**: Hovering the mouse over a region in the cartogram or conventional map causes an infotip to appear next to the mouse cursor with the region’s name, population, land area, and numeric data used to generate the cartogram.
  - **Linked brushing**: Hovering the mouse over a region in the cartogram or conventional map highlights the hovered-over region *and* the corresponding region on the other map. This feature is implemented by lightening the selected color for the region.
  - **Map-switching animation**: Using the drop-down menu above the cartogram, users may switch between the conventional map, population cartogram, and user-generated cartogram. Each time a new map is selected, the currently selected map morphs into the newly selected map through a one-second animation.
5. Users may export or share their generated cartogram by clicking the relevant buttons at the bottom of the page. Cartograms may be downloaded in the Scalable Vector Graphics (SVG) format for inclusion in a report or presentation as a figure, or in GeoJSON format for import into GIS software. Users may also opt to share their generated cartogram on popular social media sites through a unique link.

**Table 2:** Summary of automated contiguous cartogram generation software tools currently available [9, 10, 12, 17, 18, 27].

Generation Tool	Application Type	Geographic Data Source	Numeric Data Source	Interactive Analysis Tools
go-cart.io	Web application	82 available geographies from drop-down menu	CSV upload or manual entry	Yes
Cartogram Utility for ArcGIS	ArcGIS plugin	ESRI shapefile	ESRI shapefile or spreadsheet	Yes
fBlog	Web application	2 available geographies	Manual entry	No
MAPresso	Web application (obsolete)	ESRI shapefile	ESRI shapefile	Yes
ScapeToad	Desktop application	ESRI shapefile	ESRI shapefile	No
F4Carto	Desktop application (Windows only)	ESRI shapefile	ESRI shapefile	No

## Methods

### Cartogram software

When evaluating the usability of a software system, Lewis [23] recommends performing norms-based evaluations, where software system usability is evaluated against standards for usability generally applicable to software systems, and competitive evaluations. Norms-based evaluations are insufficient by themselves because they may be unduly harsh or lenient based on the category of software being considered. To conduct a competitive evaluation of go-cart.io’s usability, we performed a survey of similar cartogram generation tools. At the time of the experiment, the fBlog Online Cartogram Tool was the only such tool that is web-based and does not require users to download any software program or browser plugin.

**Fig 3:**
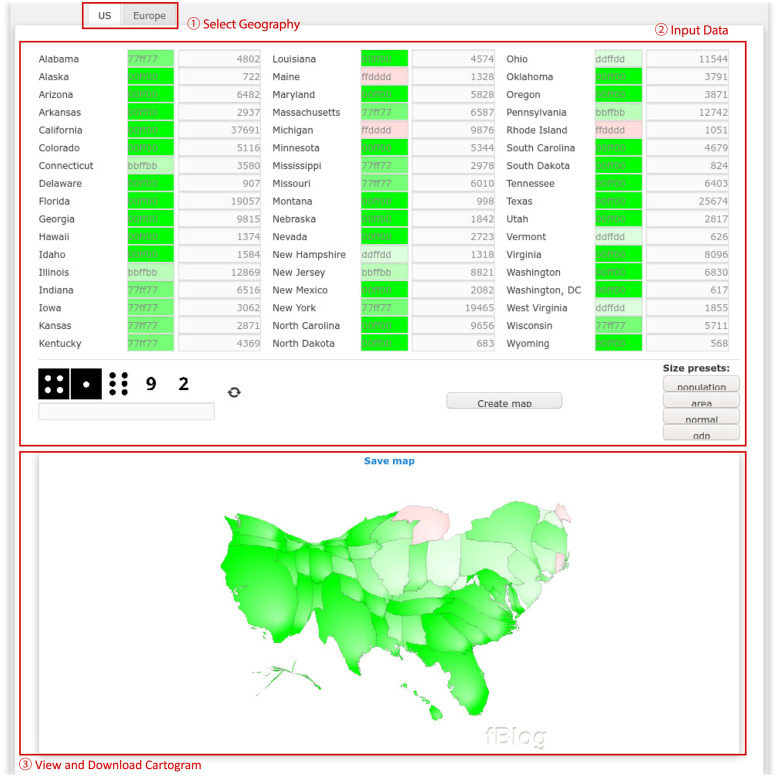
Screenshot of the fBlog Online Cartogram Tool user interface. Important interface elements are highlighted according to their functionality. Numbers in red circles indicate the sequence of steps required to create a cartogram (see main text for details). Reprinted from [10] under a CC BY license, with permission from Korneel van den Broek, original copyright 2012.

Fig 3 shows a screenshot of the fBlog user interface, which has a more linear layout than go-cart.io. To generate a cartogram, users must:

1. Select whether they would like to generate a cartogram of the United States or Europe.
2. Input numeric data and colors for each region of the selected geography in the text boxes on the page. Alternatively, users may choose from a few preset numeric data sets, including population and gross domestic product, by clicking the appropriate button at the bottom-right of the numeric input section.
3. Fill in the captcha and click “Create Map”. The cartogram image may be previewed in the browser and downloaded in Portable Network Graphics (PNG) format; however, no interactive analysis tools are provided.

### Tasks

The experiment comprised cartogram generation and analysis tasks. During the generation tasks, participants were instructed to generate a cartogram of a provided data set using one of the two generation tools (i.e., go-cart.io or fBlog). Data sets provided for both tools were for the United States because this is one of two maps available on both generation tools. A different data set was used for each generation tool to avoid a learning effect upon subsequent generation and analysis tasks. Both data sets involve agricultural data by state. To ensure similar difficulty of the analysis tasks, both datasets had a similar order of magnitude (between 10^8^ and 10^10^ USD) and were strongly correlated (*r* = 0.947). During the go-cart.io generation task, participants generated a cartogram of 2018 agricultural output by state [1], while for the fBlog generation task participants generated a cartogram of 2017 crop sales by state [28]. The usage of similar data sets helped equalize the difficulty of the subsequent analysis tasks. Additionally, we anticipated that most participants would be unfamiliar with these data sets, thereby reducing the likelihood of them relying on their own knowledge of the data sets to complete the analysis tasks.

Participants could not ask the experiment supervisor for help during generation tasks, but they were allowed to reference a written tutorial provided by each generation tool on its website. While the fBlog tutorial focuses on how to choose good data and colors for a cartogram, the go-cart.io tutorial provides step-by-step instructions for generating a cartogram once you already have a data set. Screenshots of both tutorials are available as online supplemental material on the publisher’s website. If participants could not complete a generation task, they were allowed to skip it and proceed to the tasks for the next generation tool.

Upon completing a generation task, participants were presented with the cartogram they generated and a correct reference cartogram. Participants were asked to compare the two by eye. If they found the two were *not* identical, participants could reattempt the generation task to correct their mistakes. Otherwise, they proceeded to a set of analysis tasks.

**Fig 4:**
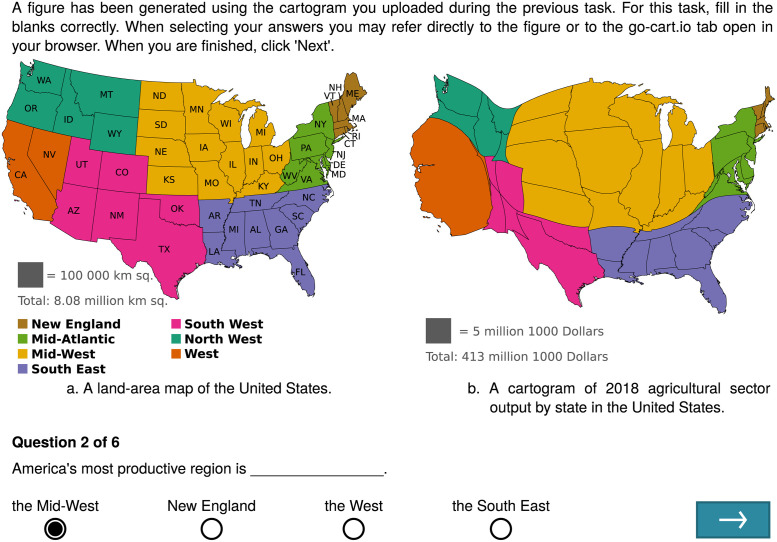
Example cartogram analysis task for go-cart.io. The example task contains a figure created from a correctly completed generation task. The figure, question, and answer options appeared on the same screen.

Analysis tasks were designed to simulate how cartograms are used as a visual aid in reports and presentations. For each analysis task, a static figure was generated from the participant’s cartogram that resembled a figure in a report. Fig 4 depicts an example cartogram analysis task and generated figure. Using the figure or any interactive analysis features provided by the generation tool, participants answered one multiple choice question about the data set. The questions in the analysis tasks were loosely inspired by the cartogram task taxonomy adopted by Nusrat, Alam, and Kobourov [2]. All analysis tasks given to participants during the experiment are available in the supplemental online material to this article.

### Participants

We recruited 35 participants to complete the experiment from August 11th to December 18th, 2020. All participants were university students and staff. A departmental ethics review committee at Yale-NUS College approved this research (case number 00016352), and the Institutional Review Board at the National University of Singapore issued a statement of concurrence for the study. Participants were recruited to the study through the college’s online research participation system. All participants provided informed consent in written form. The majority of participants reported being at least somewhat familiar with computer graphics (20 participants), spreadsheet software (28 participants), and cartograms (20 participants). Half of the participants (17) also indicated that they would at least generally look up unfamiliar locations on a map. The participants’ age ranged from 18 to 28 (mean 20.8; standard deviation 1.86), and their gender was evenly distributed (17 female; 16 male; 2 other). All participants received the equivalent of US$7.40 in local currency or one hour of research credit for a college course as compensation for participation.

We administered the Ishihara color blindness test to all participants because completing the analysis tasks required participants to distinguish between map regions by color. Three participants made at least one error during the test. However, the responses of these participants did not differ significantly from the others; thus, we included them in the data analysis.

We acknowledge that the exclusive participation of university students and staff may primarily represent a demographic of digital natives with higher education, potentially introducing bias to the results. However, the tasks in our study do not require specialized computer skills and are accessible to adolescents and adults with normal vision, including those who wear eyeglasses or contact lenses. Therefore, we believe that our results are broadly applicable to the typical user group of cartogram generation software.

### Procedure

Participants completed the experiment remotely over Zoom using a single screen. Each participant’s screen was recorded during the session so that their interactions with the generation tools could be analyzed later in more detail. We used Qualtrics XM to display the experiment tasks and collect participants’ answers. The experiment comprised four parts:

1. **Introduction:** Participants first watched a short introductory video giving a brief overview of cartograms and a description of experiment tasks. They could ask the experiment supervisor for clarification at any time. The video is included in the supplemental online material to this article.
2. **Preliminary questions:** Participants answered demographic questions about age, gender, and level of education. Then, they indicated their familiarity with computer graphics, maps, cartograms, and spreadsheet software on a 5-point Likert scale. Finally, participants completed the Ishihara color blindness test.
3. **Cartogram tasks:** Participants completed one cartogram generation task and six analysis tasks for each generation tool. After each set of tasks, participants indicated the extent to which they relied on the tutorial provided by the generation tool. If they did indicate that they relied on this tutorial, they were also required to indicate the helpfulness of the tutorial on a 5-point Likert scale. Finally, participants indicated how much they relied on the following sources when completing the analysis tasks:
  - The numbers in the data set table.
  - The cartogram they generated.
  - The interactive analysis tools provided by the generation tool.
  - Their own knowledge of the data set.
4. **Usability survey:** Participants completed an SUS questionnaire for go-cart.io and fBlog. They then left written, free-form feedback about their experience with both web tools.

### Design

We adopted a within-subject experiment design. Participants completed one trial for each generation tool. The order in which participants encountered the tools was treated as a blocking factor: 18 participants completed the go-cart.io trial before the fBlog trial, and 17 participants completed the fBlog trial before the go-cart.io trial. Each trial consisted of one generation task followed by six analysis tasks.

### Hypotheses

Prior to the experiment, we anticipated that features of go-cart.io and fBlog would impact how participants rated their relative usability. Our hypotheses were as follows:

#### Numeric data input

While go-cart.io allows users to quickly fill out and upload a CSV spreadsheet to input numeric data, fBlog requires users to enter data for each map region manually in its interface.

- **H1**: Participants would find go-cart.io more usable than fBlog because of go-cart.io’s option to upload numeric data as a CSV file.

#### Interactive analysis tools

While go-cart.io provides interactive analysis tools for generated cartograms (e.g., infotips), fBlog provides none. Duncan et al. [29] conducted an evaluation of the interactive analysis tools in go-cart.io and found them to improve performance during cartogram reading tasks.

- **H2**: Participants would find go-cart.io more usable than fBlog because go-cart.io’s interactive analysis tools will aid them in completing the cartogram analysis tasks.

#### User interface layout

While fBlog has a linear top-to-bottom user interface layout, go-cart.io provides no clear indication of where to start and how to proceed with cartogram generation.

- **H3**: Participants would find fBlog more usable than go-cart.io because its user interface layout clearly indicates how users should proceed.

### Data analysis

For analyzing generation task times, we excluded all attempts that resulted in incorrect cartograms (i.e., at least one area or one color differed from the desired output). Because the generation task times, conditioned on generating correct cartograms, were not normally distributed, we used the non-parametric paired Wilcoxon ranked-sign test to compare generation task times between generation tools. For this and other tests, we considered *p*-values as significant if they are less than 0.05, and we also computed confidence intervals. We adopted the method developed by Bauer [30] to estimate the 95% confidence interval of the pseudomedian difference in generation task times between the two tools.

For rating participants’ accuracy on the generation tasks, we analyzed mistakes made by entering the wrong color and wrong numeric data for each region separately as well as the total number of errors of all types made by each participant. Similarly, for the analysis tasks, we analyzed the error rates for each task as well as the number of analysis tasks performed correctly for each generation tool. We considered an analysis task to be performed correctly only if all parts of the task were completed correctly. A permutation test with 10000 random simulations was used to determine the effect of generation task accuracy on the number of correct analysis tasks for each generation tool.

For the SUS, we computed the score for each participant using the standard methodology presented by Brooke [22]. We used Cronbach’s alpha with an acceptability range of 0.7 < *α* < 0.95, as recommended by Lewis [23] to evaluate the internal consistency of the SUS questions as applied to this experiment. Because SUS scores were approximately normally distributed, we used a paired *t*-test to evaluate the effect of generation tool on mean SUS score. We also used Welch’s unequal variances *t*-test to evaluate the effect of generation task completion on mean SUS score for each tool.

Finally, we analyzed whether the hypotheses we presented in the previous section are supported. For H1, we used Spearman’s *ρ*, which evaluates how well a monotonic function describes the relationship between two numeric variables, to detect if there is a positive correlation between participants’ indicated familiarity with spreadsheet software and SUS score for go-cart.io. We adopted the bootstrapping method presented by Bishara and Hittner [31] to compute 95% confidence intervals for *ρ*. For H2, we also used Spearman’s *ρ* to see if there was a positive correlation between participants’ indicated reliance on go-cart.io’s interface to complete the go-cart.io analysis tasks and SUS score for go-cart.io. For H3, we considered participants’ reliance on the tutorial provided by the generation tool during the tool’s generation task as an indication that the tool’s interface is unclear. We used a permutation test with 10000 simulations to determine if reliance on the tutorial was significantly higher for go-cart.io than for fBlog.

## Results

### Generation tasks

#### Completion

Most participants completed both generation tasks. 32 out of 35 participants finished the generation task with fBlog, while 26 participants finished the generation task with go-cart.io; 23 (i.e., 65% of the participants) completed both generation tasks. One participant was not able to complete the go-cart.io generation task due to a technical error with the go-cart.io web application, and another participant mistakenly used fBlog to complete the go-cart.io generation task. Data from these two participants have been excluded from the remaining analysis.

#### Duration

**Fig 5:**
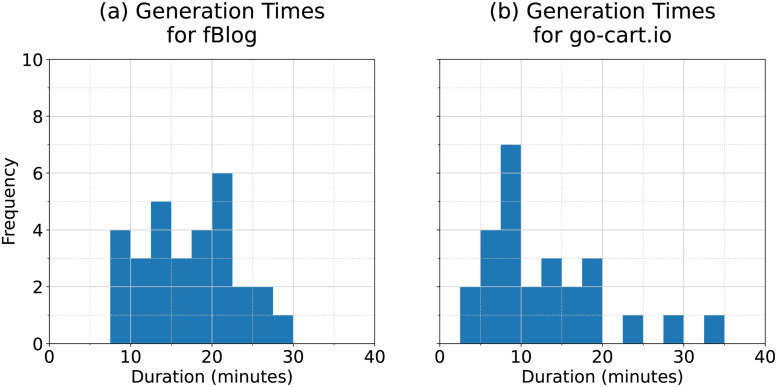
Generation task duration by generation tool. Distribution of duration in minutes taken by participants to complete both generation tasks.

Median generation task duration for fBlog, conditioned on generating the correct cartogram, was 17.2 minutes versus 10.2 minutes for go-cart.io (95% confidence interval for pseudomedian difference in minutes: [0.442, 8.77]). Fig 5 shows the distribution of generation task duration for both tools. The distribution of duration for fBlog was approximately uniform, with a minimum time of 7.62 minutes and maximum time of 28.12 minutes. By contrast, the distribution of duration for go-cart.io was right-skewed, with a minimum time of 3.70 minutes and maximum time of 34.45 minutes. The difference in pseudomedian duration between the two tools was statistically significant (*p* = 0.038).

#### Accuracy

Most participants who finished the generation tasks completed these tasks accurately. 69.2% of the participants who completed the go-cart.io generation task completed it with perfect accuracy, while this number was 50% for the fBlog generation task. Table 3 provides a breakdown of the accuracy and error rates for both fBlog and go-cart.io generation tasks. Overall, participants’ accuracy in entering state areas was high across both tasks. 92.3% of the participants completing the go-cart.io generation task entered the areas of all 48 contiguous U.S. states and Washington D.C. correctly, while 81.2% of the participants completing the fBlog generation task entered all depicted region areas correctly. For both tasks, accuracy of region colors was lower. 69.2% of the participants who completed the go-cart.io generation task entered all region colors correctly, while only 56.3% did so for the fBlog generation task.

**Table 3:** Generation task accuracy by generation tool. Accuracy and error rates for region areas and colors in the (a) fBlog and (b) go-cart.io generation tasks. “With Error” refers to the proportion of participants making at least one error in entering the area or color of a map region.

**(a) fBlog**			
Colors		Areas	
		Accurate	With Error
	Accurate	0.500	0.063
	With Error	0.313	0.125
**(b) go-cart.io**			
Colors		Areas	
		Accurate	With Error
	Accurate	0.692	0.000
	With Error	0.231	0.077

**Fig 6:**
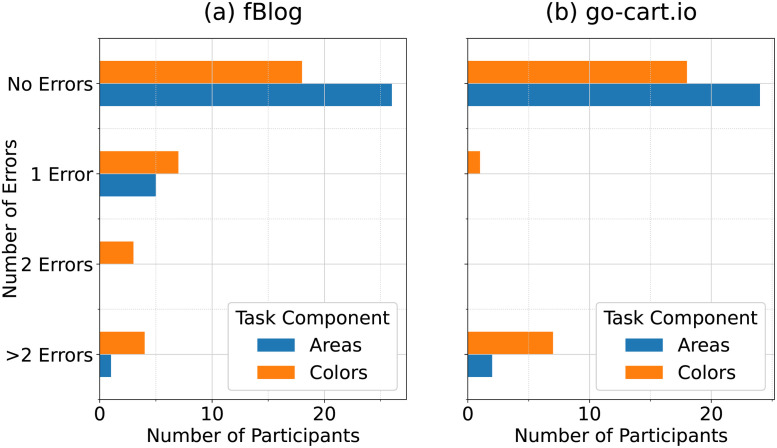
Generation task errors by generation tool. Distribution of the number of area and color errors made by participants during the (a) fBlog generation task and (b) go-cart.io generation task.

Among participants who made errors during the fBlog generation task, the number of errors was usually small. Fig 6 shows the distribution of the number of area and color errors for both generation tasks. For the fBlog generation task, 5 out of the 6 participants who made an area error made only one such error. Similarly, 10 out of the 14 participants who made a color error on the fBlog task made at most two such errors.

However, the opposite is true for the go-cart.io generation task. Both participants who made an area error made at least three such errors, and 7 out of the 8 participants who made a color error made at least three such errors.

#### Reliance on and helpfulness of tutorial

**Fig 7:**
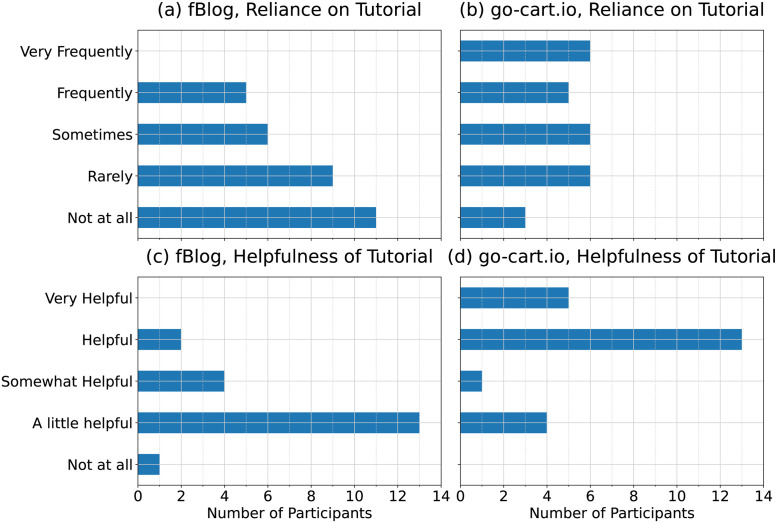
Self-reported reliance and helpfulness of generation tool tutorials. Panels (a) and (b) show how often participants indicated that they relied on the written tutorials provided by fBlog and go-cart.io, respectively, during each generation task. For participants who indicated that they relied on the tutorial for a generation tool at least “Rarely”, panels (c) and (d) show how helpful participants rated the respective tutorials for fBlog and go-cart.io.

Participants indicated lower reliance on the tutorial during the fBlog generation task than during the go-cart.io generation task. Fig 7 provides an overview of participants’ indicated reliance on and helpfulness of the tutorials during both generation tasks. Considering participants’ responses on an interval scale from 0 (“Not at all”) to 4 (“Very frequently”), mean reliance on the tutorial was found to be 1.03 points higher for go-cart.io than for fBlog (95% confidence interval [0.491, 1.58]). Mean reliance on the tutorial for fBlog was 2.19, while it was 1.16 for fBlog. This difference is significant (*p* = 0.002).

Among participants who relied on a tutorial, the mean helpfulness of the go-cart.io tutorial was 1.48 points higher than the mean helpfulness of the fBlog tutorial (95% confidence interval [0.962, 1.98]), on an interval scale from 0 (“Not at all”) to 4 (“Very helpful”). Mean helpfulness was 1.35 for the fBlog tutorial and 2.83 for the go-cart.io tutorial. This difference is also significant (*p* < 0.001).

### Analysis tasks

#### Error rates

**Fig 8:**
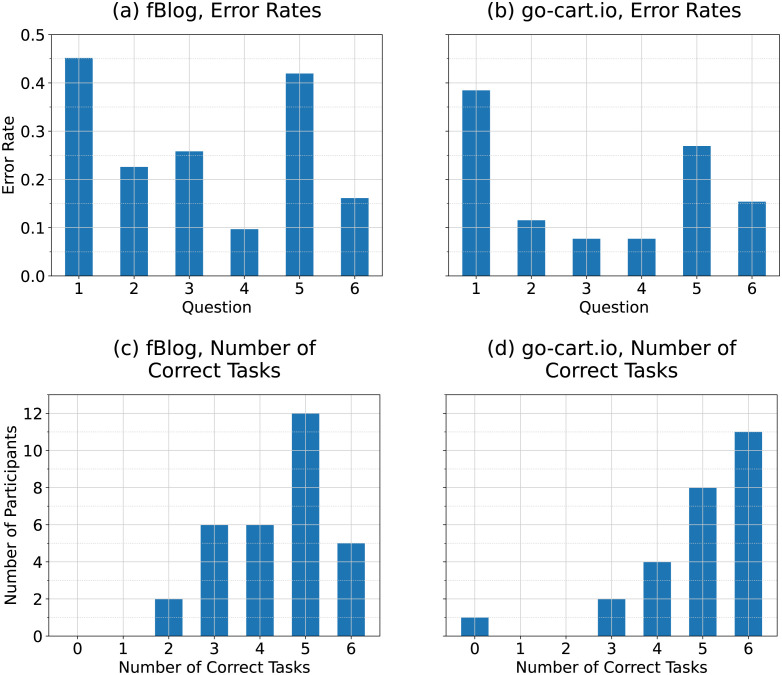
Participants’ performance on analysis tasks. Panels (a) and (b) show error rates by analysis task question for fBlog and go-cart.io, respectively. Panels (c) and (d) show the distribution of the number of correct tasks for fBlog and go-cart.io, respectively.

Participants found most analysis tasks for both fBlog and go-cart.io to have moderate difficulty, with some tasks being unexpectedly very difficult. Fig 8 provides an overview of participants’ performance on the analysis tasks. The first analysis task for both generation tools had the highest error rates (0.452 for fBlog and 0.385 for go-cart.io). This may have been the partial result of the first analysis task having two parts for both generation tools (participants had to name the largest and second-largest region), while all other analysis tasks had only one part.

#### Effect of generation tool

For both generation tools, a majority of the participants completed over half of the analysis tasks correctly (74.2% for fBlog and 88.5% for go-cart.io).

#### Effect of generation task accuracy

Participants who completed the fBlog generation task without error completed on average 0.467 (95% confidence interval [−0.067, 1.00]) more fBlog analysis tasks correctly than those who made at least one error of any type during the generation task. However, fBlog generation task accuracy did not have a significant effect on the number of fBlog analysis tasks completed correctly (*p* = 0.145).

Participants who completed the go-cart.io generation task without error completed on average 0.611 (95% confidence interval [−0.795, 2.03]) more go-cart.io analysis tasks correctly than participants who made at least one generation task error. The accuracy of go-cart.io generation tasks did not have a significant effect either on the number of go-cart.io analysis tasks completed correctly (*p* = 0.147).

#### Participants’ indicated methodology

**Fig 9:**
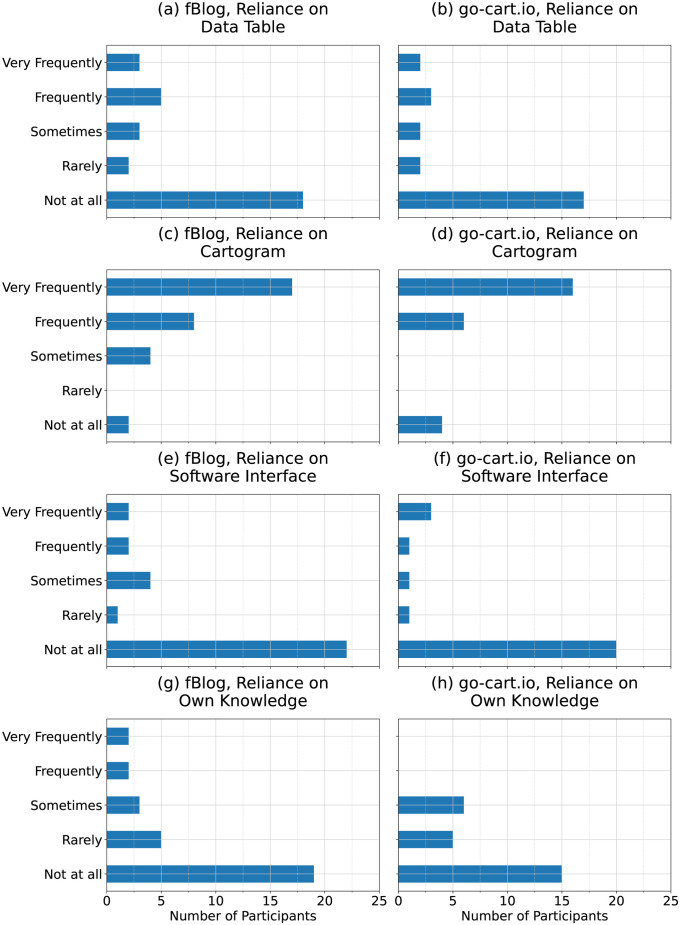
Participants’ indicated methodology for completing analysis tasks. Participants’ indicated reliance on the data table [panels (a) and (b)], generated figure with cartogram [panels(c) and (d)], software interface [panels (e) and (f)], and their own knowledge [panels (g) and (h)] while completing the analysis tasks for fBlog and go-cart.io.

Most participants relied on the figure created from their cartogram during the generation tasks for both generation tools. Fig 9 provides an overview of the sources of information participants indicated they relied on while completing the analysis tasks. 80.6% of the participants who completed the fBlog analysis tasks reported relying on the generated cartogram figure frequently or very frequently, while 84.6% who completed the go-cart.io analysis tasks indicated the same.

A minority relied on the numbers in the data table or their own knowledge to complete the analysis tasks. Only 35.5% of those completing the fBlog analysis tasks reported relying on numbers in the data table more than rarely, and 22.6% relied on their own knowledge more than rarely. Similarly, 26.9% of the participants completing the go-cart.io analysis tasks indicated that they relied on the numbers in the data table more than rarely, and 23.1% relied on their own knowledge more than rarely.

Reported reliance on the generation tool interface, including interactive analysis tools, was also low. Only 24.4% of those completing the fBlog analysis tasks reported relying on the fBlog interface, while 19.9% indicated the same for go-cart.io.

### System Usability Scale score

The SUS is a highly reliable measure of perceived usability for both fBlog (*α* = 0.860) and go-cart.io (*α* = 0.923).

The mean SUS score was 18.2 points higher for go-cart.io than for fBlog (95% confidence interval [6.78, 29.6]). While the mean SUS score for fBlog was 47.1 (standard deviation 19.4), the mean SUS score for go-cart.io was 65.3 (standard deviation 23.0). The difference in mean SUS score for fBlog and go-cart.io was significant (*p* = 0.003).

Completion of the generation task for fBlog and go-cart.io was associated with a higher mean SUS score. The mean fBlog SUS score for participants failing to complete the fBlog generation task was 30; however, for those who completed this task, the mean SUS score was 48.0 (95% confidence interval of difference in means: [7.93, 28.1]). Similarly, while the mean go-cart.io SUS score was 38.6 for participants who did not complete the go-cart.io generation task, the mean SUS score was 72.5 for those who did complete this task (95% confidence interval of difference in means [16.7, 51.1]). For both fBlog (*p* = 0.003) and go-cart.io (*p* = 0.001), completion of generation task had a significant effect on mean SUS score.

Participants’ indicated familiarity with spreadsheet software was not significantly correlated with the SUS score for fBlog (*ρ* = 0.318, 95% confidence interval [−0.03, 0.596]) or go-cart.io (*ρ* = 0.014, 95% confidence interval [−0.331, 0.355]. Reliance on the generation tool interface, including any interactive analysis tool, during the analysis tasks was not significantly correlated with SUS score for fBlog (*ρ* = 0.190, 95% confidence interval [−0.176, 0.510]) or go-cart.io (*ρ* = 0.236, 95% confidence interval [−0.166, 0.571]) either.

### Written participant feedback

#### fBlog

Participants reported using fBlog to be “troublesome” and “tedious” due to the manual numeric data-entry method. They also indicated that they would have preferred a spreadsheet upload option, as implemented in go-cart.io, or the ability to copy tabular data directly into the web interface.

#### go-cart.io

Several participants indicated that they found the go-cart.io interface to be aesthetically pleasing. While a few participants wrote that the generation tool was easy to use, most indicated that they faced issues using the spreadsheet upload feature to input numeric data for the generation task. Participants complained that the instructions for formatting and saving the spreadsheet were unclear. Some participants were unaware that only CSV spreadsheet files could be uploaded and expressed frustration that Microsoft^®^ Excel spreadsheets could not be uploaded. Many of the participants who eventually succeeded indicated that they relied heavily on go-cart.io’s written tutorial and had to read it carefully. Participants who gave up trying to use the spreadsheet upload feature and instead used the pop-up editing interface [shown in panel (b) of Fig 2] complained that entering map region colors was difficult because color codes could not be pasted into the editing interface.

### Hypotheses

#### H1 is rejected: go-cart.io’s numeric data input is not more usable than that of fBlog

While several participants indicated in their written feedback that they preferred go-cart.io’s spreadsheet upload option to fBlog’s manual input method, most participants struggled to utilize the former method. Additionally, we did not find a significant relationship between participants’ familiarity with spreadsheet software and their perceived usability of go-cart.io. For these reasons, H1 is rejected.

#### H2 is rejected: go-cart.io’s interactive analysis tools played no significant role for usability

Few participants made use of the interactive analysis tools provided by go-cart.io to complete the go-cart.io analysis tasks, and there was no significant correlation between increasing reliance on go-cart.io’s interactive analysis tools and SUS score. For these reasons, H2 is rejected.

#### H3 is partially supported: go-cart.io’s user interface layout leads to more frequent referencing of tutorials than that of fBlog

Although mean SUS score was significantly lower for fBlog than for go-cart.io, participants relied on the tutorial to complete the go-cart.io generation task significantly more than during the fBlog generation task. This greater reliance implies that more participants were unable to use go-cart.io without guidance than fBlog. H3 is thus partially supported.

## Discussion

A majority of the participants were able to complete the generation and analysis tasks for go-cart.io with reasonable accuracy. Participants who did make errors on the go-cart.io generation task were more likely to have made many more errors than for the fBlog generation task, where those who made errors generally made only one or two. We hypothesize that the differing numeric data and color entry methods of the two tools account for this difference. For the go-cart.io task, participants were able to copy numeric data and region colors as columns and paste them into a CSV spreadsheet for upload. This method of data entry means that likely sources of error, such as transposition of spreadsheet columns, would lead to many errors in the generated map. By contrast, fBlog’s manual entry method caused each typo or incorrect pasting to affect only one region.

The time savings from using the spreadsheet upload feature as compared to manual entry of numeric and color data for each region were substantial, accounting for the significant difference in median generation time between fBlog and go-cart.io. To help users detect when they have made errors in the spreadsheet they upload to go-cart.io, the application shows uploaded numeric data in pie chart form and asks users to confirm that the data are correct and appropriate for a cartogram before proceeding to the generation phase. Panel (c) of Fig 2 shows the pie-chart display.

Yet, these time savings did not translate into a high usability rating of go-cart.io by participants. The mean SUS score of 65.6 for go-cart.io is slightly lower than the mean SUS score of 65.7 for desktop GIS software tools found by Davies and Medyckyj-Scott [16]. Like the respondents to their 1994 survey, participants in this experiment complained that go-cart.io was unintuitive to use and had poor error messages. In the following sections, we make recommendations to improve the usability of go-cart.io as a web-based GIS tool. We believe these recommendations will also be informative for the development of other web-based cartogram generation tools.

### Recommendations

#### Data entry

Entering the numeric data and color for each map region during the go-cart.io generation task proved to be one of the most difficult tasks for participants during the experiment. A variety of factors are responsible for making this task more difficult than anticipated.

First, while go-cart.io only accepts CSV-format spreadsheets for upload, several participants were unaware of the difference between spreadsheet formats and erroneously assumed they could upload Excel-format spreadsheets.

- **Recommendation 1:** Due to the format’s popularity, web-based cartogram generation tools should accept Excel-format spreadsheet files in addition to CSV-format spreadsheet files. Support for additional file formats, such as Open Document Format Spreadsheet (ODS) and JavaScript Object Notation (JSON), is also desirable.

**Fig 10:**
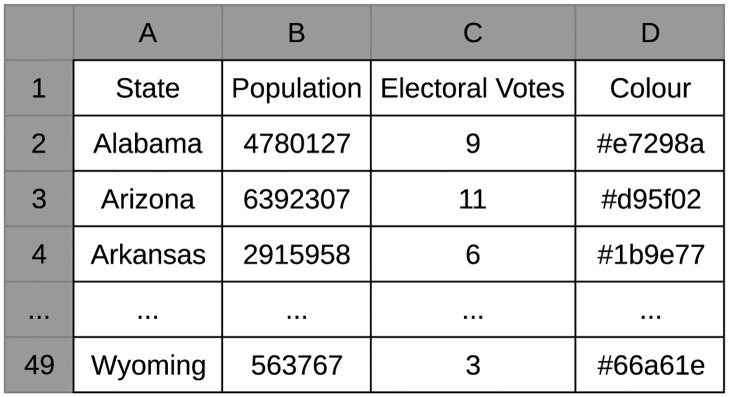
Excerpt of go-cart.io CSV spreadsheet template for the “Conterminous United States” map. This template must be edited and uploaded by users to generate a cartogram of their own data set. The “Population” column is only for reference, while users must overwrite the third and fourth columns with their numeric and color data, respectively, for each region.

Secondly, go-cart.io requires the numeric and color data for each region in the uploaded spreadsheet to be organized in a very particular way. Fig 10 shows a spreadsheet template for the “Conterminous United States” map that participants had to edit, save, and reupload to generate their cartogram using the spreadsheet upload feature. In order for go-cart.io to recognize their data, participants were required to delete the third and fourth columns and replace them with the numeric and color data, respectively, given during the generation task. If participants replaced the “Population” column instead, or inserted another column before or after the “Colour” column, their data were either silently ignored, or a nondescript error message “There was a problem reading your CSV file” was displayed. No participant was able to successfully complete the go-cart.io task using the spreadsheet upload feature without referencing the tutorial, and most made several attempts while referencing the tutorial before they were successful.

- **Recommendation 2:** Web-based cartogram generation tools should attempt to automatically determine which columns in the uploaded spreadsheet contain the numeric information for target areas (e.g., population) and other visual variables (e.g., color) for each region.
- **Recommendation 3:** If the cartogram tool is unable to parse the uploaded spreadsheet, it should provide a descriptive error message with hints about possible fixes (e.g., “It looks like you have uploaded a spreadsheet for Austria, even though you have selected a map of the United States.”).

Finally, although the spreadsheet upload feature is the preferred data-entry method for go-cart.io, the pop-up editing interface [shown in panel (b) of Fig 2] remains an important alternative data-entry method. Several participants used this interface after they were unable to successfully use the spreadsheet upload feature. However, the pop-up editing interface suffers from several usability challenges. While it is styled to look like a spreadsheet, the interface does not support basic spreadsheet data-entry methods. Entire columns cannot be copied and pasted, only individual cells. Additionally, the implementation of the color column using the HTML color input element means that color codes cannot be pasted at all on some browsers.

- **Recommendation 4:** Web-based cartogram generation tools should include a pop-up editing interface as an alternative to uploading a spreadsheet. The interface should support copying and pasting entire columns to and from the clipboard.

#### Interactive analysis tools

We hypothesized that participants would rely on the interactive analysis tools (infotip, linked brushing, and morphing animations) provided by go-cart.io because Duncan et al. [29] showed that these tools improve performance during certain cartogram tasks. However, this hypothesis (H2) was rejected. Multiple factors may account for participants ignoring the interactive features. First, many of the analysis tasks were relatively simple. Duncan et al. [29] found that interactive analysis tools are not beneficial for simple tasks; thus, participants in this experiment may have avoided using these tools for some tasks because they were unnecessary.

Secondly, while the go-cart.io website itself provides interactive analysis tools, the SVG images that it produces for export are static and do not support any interactivity. Because these SVG images were used to generate the figure for the go-cart.io analysis tasks during the experiment, the figure did not support interactivity either. To make use of the interactive analysis tools provided by go-cart.io, participants had to switch back to the go-cart.io tab in their browser during the experiment. Thus, we believe that participants were likely disinclined to use go-cart.io’s interactive analysis tools because they were not readily accessible.

- **Recommendation 5:** Web-based cartogram generation tools should include interactive features for analysis (e.g., infotips). The generation tools should support embedding cartograms with interactive features on other websites.

#### User interface layout

While participants complimented the aesthetics of go-cart.io’s user interface, many complained that the generation tool interface was unintuitive. Indeed, participants’ reliance on the tutorial during the generation tasks was much higher for go-cart.io than for fBlog. Having good written documentation has been shown to improve the perceived usability of a software system [16], and most participants rated the go-cart.io tutorial as helpful or very helpful. However, as a web-based tool, go-cart.io cannot expect users to read an extensive written tutorial before using the tool. If a user gets an initial impression that a web-based tool is difficult to use or unintuitive, they are likely to abandon it quickly.

**Fig 11:**
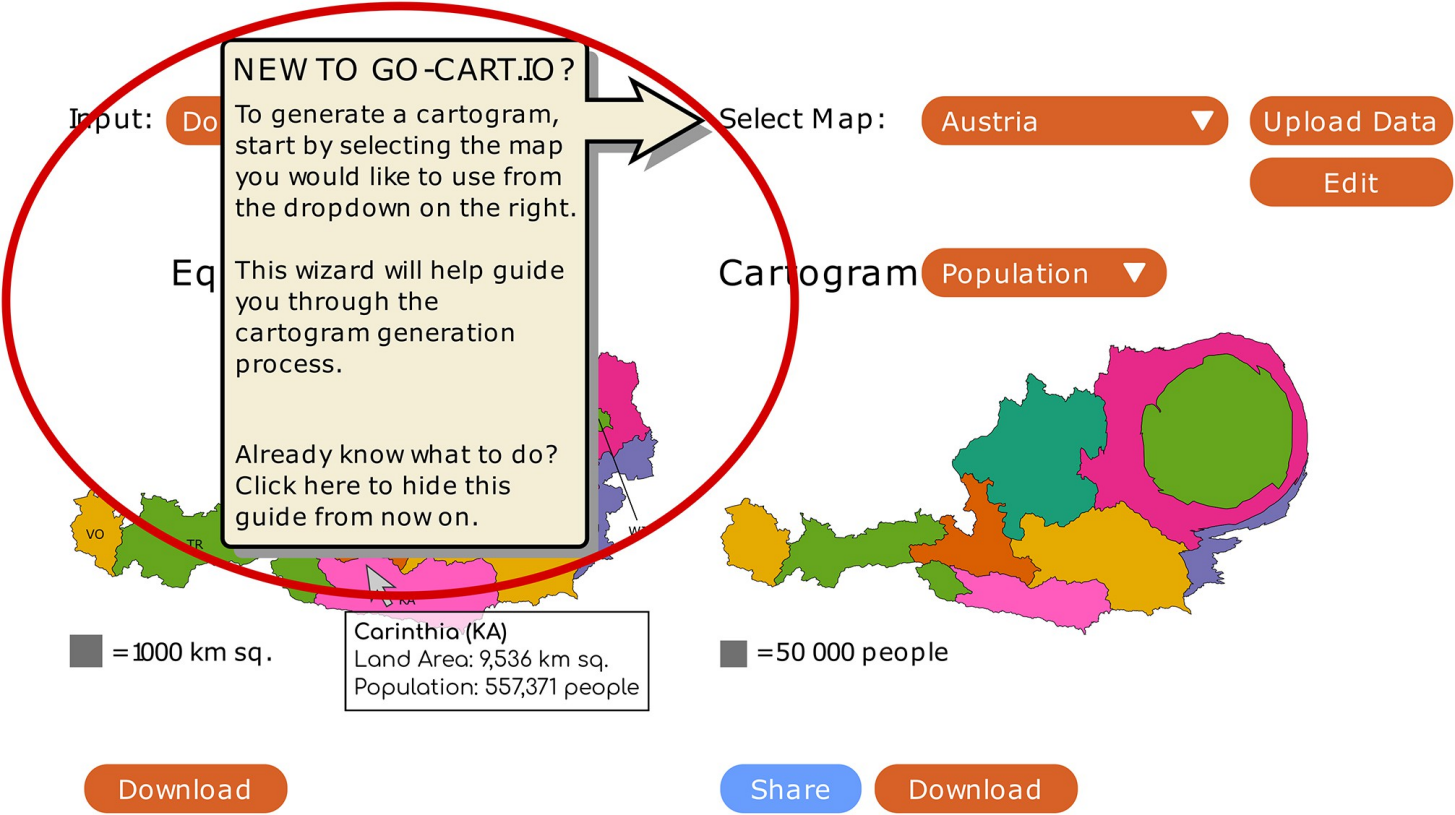
Proposed appearance of tutorial overlay. Screenshot of the go-cart.io user interface with a proposed tutorial overlay (circled in red) to improve usability.

- **Recommendation 6:** Web-based cartogram generation tools should implement a tutorial overlay that guides users through the cartogram generation process without requiring users to reference a separate written tutorial. Fig 11 depicts a proposed design for this overlay on go-cart.io.

## Conclusions

Although cartograms are becoming increasingly popular for visualizing geospatial data, cartogram generation software has historically suffered from poor usability, similar to many other GIS software. While Shi, Duncan, and Gastner [12] designed go-cart.io with the explicit goal of creating an easy-to-use web-based cartogram generation tool, the results of our experiment show that go-cart.io has poor usability in key areas such as data entry. The purpose of this study was to make concrete recommendations for future designs of web-based cartogram generation tools based on the usability concerns raised by participants.

We believe that the user-centered evaluation and comparative analysis of two web-based cartogram tools (go-cart.io and fBlog) offers practical insights by focusing on real-world usage. However, we acknowledge the limitations of our study. It primarily addressed usability and user perception, omitting in-depth technical assessments of the cartogram generation process and the quality of the generated cartograms. Additionally, a broader sample size and greater participant diversity could have enhanced the generalizability of our findings. Moreover, we could have expanded the comparative analysis to include cartogram generation tools that were not in English or not web-based.

Nonetheless, it is safe to conclude that both tools that were investigated in this study (go-cart.io and fBlog) would have benefited from applying the standard guidelines of user-centered design early on in their design [32]. We also believe that this study reveals general usability issues that are applicable to web-based cartogram generation. We hope that our recommendations will advance the usability of cartogram software so that more users will be able to produce cartograms as an alternative to traditional types of thematic maps (e.g., proportional-symbol or dot-density maps). We have informed the go-cart.io developers about our recommendations. Implementing and evaluating the effect of these recommended practices is a source of future work.

## Supporting information

S1 File Tutorial of fBlog Online Cartogram Tool. Reprinted from [10] under a CC BY license, with permission from Korneel van den Broek, original copyright 2012. Downloaded on 22 January 2022.
(PDF)

S2 File Tutorial of go-cart.io. Downloaded from https://go-cart.io/tutorial on 22 January 2022.
(PDF)

S3 File Analysis tasks performed by participants during the experiment. Each analysis task is presented alongside the figure participants would have seen while completing the task if they completed the corresponding generation task with no errors. Images for tasks involving fBlog were reprinted from [10] under a CC BY license, with permission from Korneel van den Broek, original copyright 2012.
(PDF)

S4 File List of maps preinstalled in go-cart.io. This information was retrieved from https://go-cart.io/cartogram on October 28th, 2023.
(PDF)

S1 Video Introductory video shown to participant. All participants watched this 4-minute video before the cartogram generation and analysis tasks.
(MP4)

S1 Text The data and analysis scripts that support the findings of this study are available at https://figshare.com/s/592d9e8dcf5aa933d800. (TXT)
