# Supplementary material for: Comparative evaluation of the web-based contiguous cartogram generation tool go-cart.io
Source: PLoS One. 2024 May 8;19(5):e0298192. doi: 10.1371/journal.pone.0298192 (PMC11078394; doi:10.1371/journal.pone.0298192)
Supplement: S1 File — Reprinted from [10] under a CC BY license, with permission from Korneel van den Broek, original copyright 2012. Downloaded on 22 January 2022. (PDF) [file pone.0298192.s001.pdf]

Online Cartogram Tool

Posted on **May 18, 2012** by **korneelvdbroek** **1 Reply**

I have always found good visualization of data very important. A nice way to represent data related to maps is to use cartograms. Where a normal map allows you to color each country/state with a color representing your data, a cartogram allows you to convey much more information. In a cartogram, just as for a map, you also color each region to represent data, but on top of that, you inflate/deflate the size of each region to represent some more data.

In the example on the bottom of this post, each US state is colored green/red depending on its population growth. The states are inflated to represent the total population living in the state (I used recent data from Wikipedia). The other tab has the same example but for the European countries.

With the tool at the bottom of this post you can create your own maps and cartograms. Choose a color for each state/country and pick the size to which each country needs to be rescaled, click the Create Map button and your cartogram will be made.

Usually it is best to choose an intensive property to determine the color. An intensive property is data that does not depend on the size. Population growth is an example of such data. Population growth can be 1% no matter whether we are talking about a country with a large population or a small country.
On the other hand, the size is best determined by an extensive property, a property that does depend on the size. Examples are population, area, gdp, debt, … The tool comes with some presets for the extensive properties. If you choose area as extensive variable the size of each country will be its actual area (and you will thus get a normal map that is not inflated).

To represent data as a color, I find it useful to divide the data into bins. Each bins then gets a discrete color. The color scheme used in the example pictures below are: from 00ff00 for full green, over 77ff77, bbffbb, to ddffdd for transparent/white green. For red the same idea applies: from ff0000, over ff7777, ffbbbb, to ffdddd.

If you are interested in more elaborate cartograms (e.g. the entire world), you are always welcome to contact me (leave a comment and I will get back to you). If you can provide me with an xml file in this format. The size is the inflation/deflation factor, the color tag encodes the rgba-color as a decimal. The rowname should correspond to the shapefile data from the map. I got all the shapefile map data on the wonderful website of Natural Earth.

Happy mapping!

US | Europe

Alabama	77ff77	4802	Louisiana	00ff00	4574	Ohio	ddffdd	11544
Alaska	00ff00	722	Maine	ffdddd	1328	Oklahoma	00ff00	3791
Arizona	00ff00	6482	Maryland	00ff00	5828	Oregon	00ff00	3871
Arkansas	00ff00	2937	Massachusetts	77ff77	6587	Pennsylvania	bbffbb	12742
California	00ff00	37691	Michigan	ffdddd	9876	Rhode Island	ffdddd	1051
Colorado	00ff00	5116	Minnesota	00ff00	5344	South Carolina	00ff00	4679
Connecticut	bbffbb	3580	Mississippi	77ff77	2978	South Dakota	00ff00	824
Delaware	00ff00	907	Missouri	77ff77	6010	Tennessee	00ff00	6403
Florida	00ff00	19057	Montana	00ff00	998	Texas	00ff00	25674
Georgia	00ff00	9815	Nebraska	00ff00	1842	Utah	00ff00	2817
Hawaii	00ff00	1374	Nevada	00ff00	2723	Vermont	ddffdd	626
Idaho	00ff00	1584	New Hampshire	ddffdd	1318	Virginia	00ff00	8096
Illinois	bbffbb	12869	New Jersey	bbffbb	8821	Washington	00ff00	6830
Indiana	77ff77	6516	New Mexico	00ff00	2082	Washington, DC	00ff00	617
Iowa	77ff77	3062	New York	77ff77	19465	West Virginia	ddffdd	1855
Kansas	77ff77	2871	North Carolina	00ff00	9656	Wisconsin	77ff77	5711
Kentucky	77ff77	4369	North Dakota	00ff00	683	Wyoming	00ff00	568

3 8

Create map

Size presets:
population
area
normal
gdp

Save map

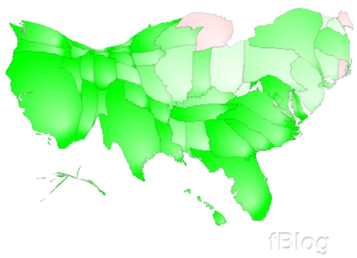

This entry was tagged **data visualization**, **map** by **korneelvdbroek**. Bookmark the **permalink**.

Comment

daniel bramatti on **November 7, 2012 at 5:31 pm** said:

Hello. You have a nice tool there! Is it possible to use it with other shapefiles? I'd love to try it with a brazilian one…

Leave a Reply

Your email address will not be published. Required fields are marked *

Comment

Name *

Email *

Website

Save my name, email, and website in this browser for the next time I comment.

Post Comment

fBlog by **Korneel van den Broek** is licensed under a
Creative Commons Attribution-NonCommercial-ShareAlike 3.0 Unported License.
Powered by WordPress
