# Supplementary material for: Comparative evaluation of the web-based contiguous cartogram generation tool go-cart.io
Source: PLoS One. 2024 May 8;19(5):e0298192. doi: 10.1371/journal.pone.0298192 (PMC11078394; doi:10.1371/journal.pone.0298192)
Supplement: S2 File — Downloaded from https://go-cart.io/tutorial on 22 January 2022. (PDF) [file pone.0298192.s002.pdf]

Cartogram Generation Tutorial

1. Select a Map

On the right-hand side of the 'Input' section, there are currently several maps to choose from. If you select a map, the display below will update to display the conventional land-area version of the map, and a population cartogram.

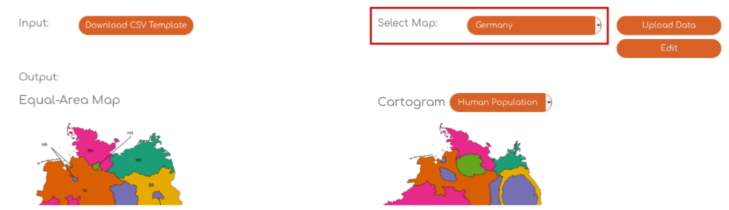

2. Fill In Your Data

The easiest wayt to fill in your data is to use a spreadsheet program like Microsoft Excel or LibreOffice Calc. Click the 'Download CSV Template' button on the left-hand side of the 'Input' section. You can open the downloaded template with the spreadsheet program of your choice.

	A	B	C	D
1	State	Population	GDP	Colour
2	Hamburg	1787408	117572	#66a61e
3	Niedersachsen	7926599	287959	#d95f02
4	Bremen	671489	33662	#7570b3
5	Nordrhein-Westfalen	17865516	691518	#e7298a
6	Hessen	6176172	279085	#66a61e
7	Rheinland-Pfalz	4052803	144308	#e6ab02
8	Baden-Wuerttemberg	10879618	493265	#d95f02
9	Bayern	12843514	594447	#7570b3
10	Saarland	995597	35300	#66a61e
11	Berlin	3520031		#7570b3
12	Brandenburg	2484826		#e6ab02
13	Mecklenburg-Vorpommern	1612362		#1b9e77
14	Sachsen	4084851		#1b9e77
15	Sachsen-Anhalt	2245470		#7570b3
16	Thueringen	2170714		#e6ab02
17	Schleswig-Holstein	2858714		#e7298a
18				

The province or state column, as well as the population column, are for reference only. You will need to insert your data into the third column, and change the header for the third column to match the name of your dataset. If you do not like the default colour scheme, you may also specify your own colour for each region by using hexadecimal colour codes.

For more information about suitable datasets and colour codes, please see the FAQ.

When you've filled in the template with your data and colour information, go back to the go-cart.io website and click 'Upload Data' in the top right corner of the website. Once you select your CSV file to upload, cartogram generation will begin. The time it takes to generate a cartogram varies, but it averages around a few seconds.

Alternatively, in a pinch you may use the built-in editing interface, which can be launched by clicking the 'Edit' button on the far right-hand side of the input section.

Germany Update

State	Population	GDP	Colour
Hamburg	1787408	117572	
Niedersachsen	7926599	287959	
Bremen	671489	33662	
Nordrhein-Westfalen	17865516	691518	
Hessen	6176172	279085	
Rheinland-Pfalz	4052803	144308	
Baden-Wuerttemberg	10879618	493265	
Bayern	12843514	594447	
Saarland	995597	35300	
Berlin	3520031	136614	
Brandenburg	2484826	69132	
Mecklenburg-Vorpommern	1612362	42783	
Sachsen	4084851	121738	
Sachsen-Anhalt	2245470	60095	
Thueringen	2170714	61907	
Schleswig-Holstein	2858714	93367	

The editor interface functions like a spreadsheet program. Again, the region name and population columns are for reference only, and cannot be changed. You will need to insert your data into the third column. You should also fill in the title of your dataset into the header for the third column.

When you are done filling in your data, click the 'Update' button at the top of the editor interface to generate your cartogram.

3. View, Download, and Share Your Cartogram

When your cartogram has finished generating, our website provides several interactive tools to help you analyze it. Hovering over each region displays detailed information about that region in a tooltip. Additionally, you may switch between your cartogram, a population cartogram, and the land area map by using the dropdown in the right hand column.

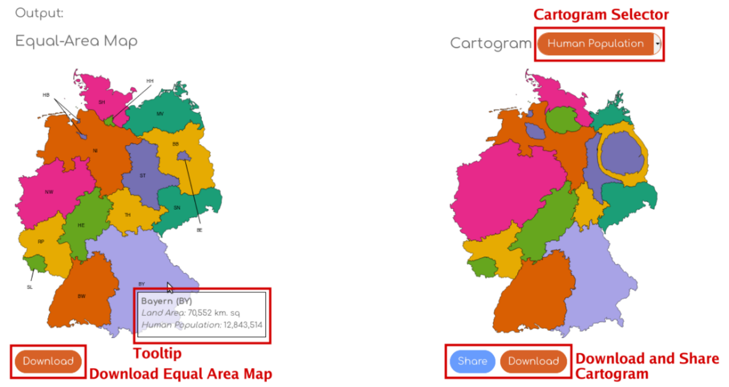

You may also download an SVG version of each of these maps by using the buttons below each map. Finally, you can also share your map on social media by using the button at the bottom of the page.

© Michael T. Gastner
michaelgastner.com
Yale-NUS College, Division of Science
16 College Avenue West,
#01-220 Singapore 138527
