# Supplementary material for: Comparative evaluation of the web-based contiguous cartogram generation tool go-cart.io
Source: PLoS One. 2024 May 8;19(5):e0298192. doi: 10.1371/journal.pone.0298192 (PMC11078394; doi:10.1371/journal.pone.0298192)
Supplement: S3 File — Each analysis task is presented alongside the figure participants would have seen while completing the task if they completed the corresponding generation task with no errors. Images for tasks involving fBlog were reprinted from [10] under a CC BY license, with permission from Korneel van den Broek, original copyright 2012. (PDF) [file pone.0298192.s003.pdf]

An evaluation of the usability of web-based contiguous cartogram generation tools: Experiment analysis tasks

January 24, 2022

This supplementary material contains all of the analysis tasks for fBlog and go-cart.io completed by participants during the experiment. Each analysis task is presented alongside the figure participants would have seen while completing the task if they completed the corresponding generation task with no errors.

1 fBlog

1.1 Task 1

A figure has been generated using the cartogram you uploaded during the previous task. For this task, fill in the blanks correctly. When selecting your answers you may refer directly to the figure or to the fBlog tab open in your browser. When you are finished, click 'Next'.

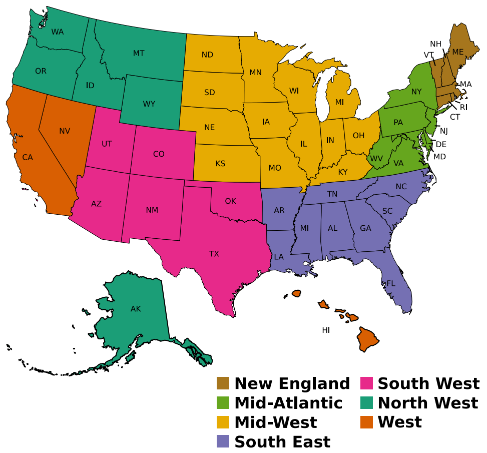

Figure 1a. A land-area map of the United States.

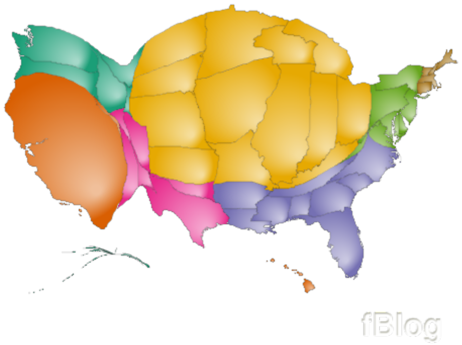

Figure 1b. A cartogram of 2017 crop sales by state in the United States.

Question 1 of 6

____(A)____ has the highest crop sales in the Mid-West, followed by ____(B)____.

	Iowa (IA)	North Dakota (ND)	Illinois (IL)	Wisconsin (WI)
A				
B				

1.2 Task 2

A figure has been generated using the cartogram you uploaded during the previous task. For this task, fill in the blanks correctly. When selecting your answers you may refer directly to the figure or to the fBlog tab open in your browser. When you are finished, click 'Next'.

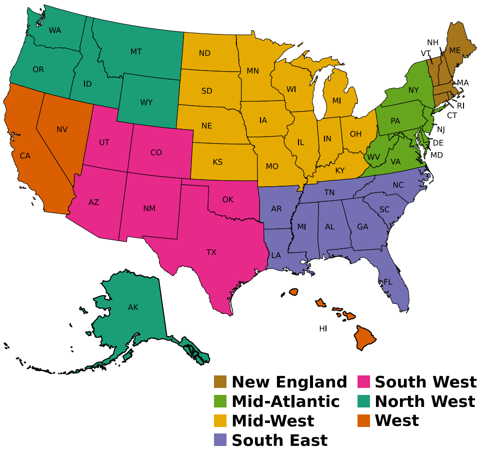

Figure 1a. A land-area map of the United States.

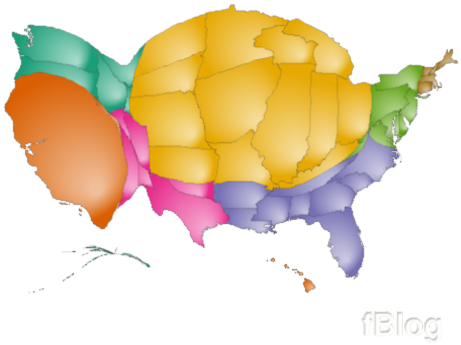

Figure 1b. A cartogram of 2017 crop sales by state in the United States.

Question 2 of 6

After the Mid-West, ____________ is the region with the highest crop sales.

A. the Mid-Atlantic
B. New England
C. the West
D. the South East

1.3 Task 3

A figure has been generated using the cartogram you uploaded during the previous task. For this task, fill in the blanks correctly. When selecting your answers you may refer directly to the figure or to the fBlog tab open in your browser. When you are finished, click 'Next'.

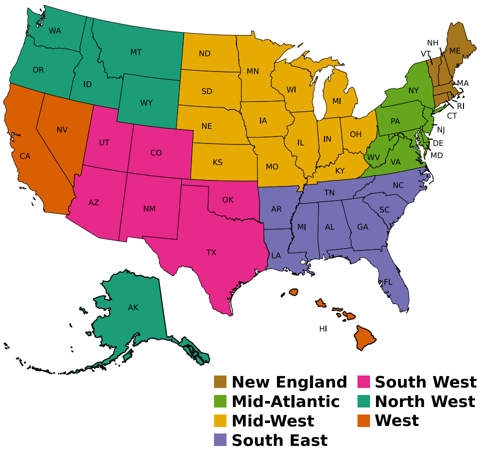

Figure 1a. A land-area map of the United States.

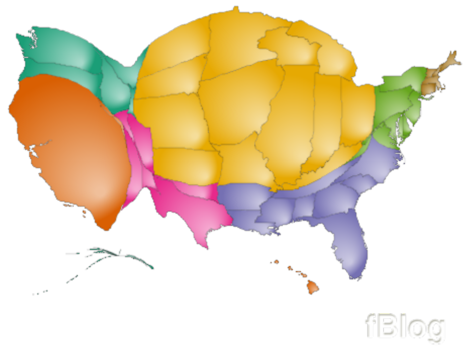

Figure 1b. A cartogram of 2017 crop sales by state in the United States.

Question 3 of 6
The crop sales of the Mid-Atlantic are ____________ those of the North West.

A. less than
B. approximately equal to
C. greater than

1.4 Task 4

A figure has been generated using the cartogram you uploaded during the previous task. For this task, fill in the blanks correctly. When selecting your answers you may refer directly to the figure or to the fBlog tab open in your browser. When you are finished, click 'Next'.

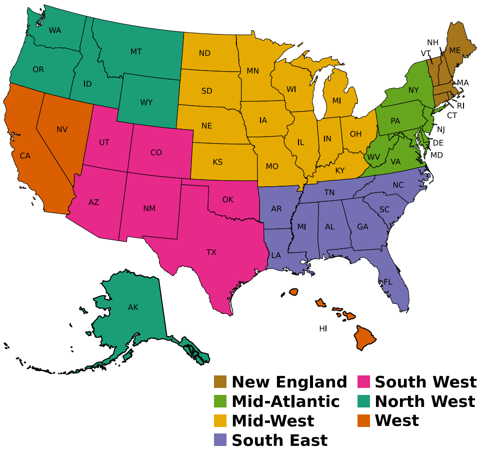

Figure 1a. A land-area map of the United States.

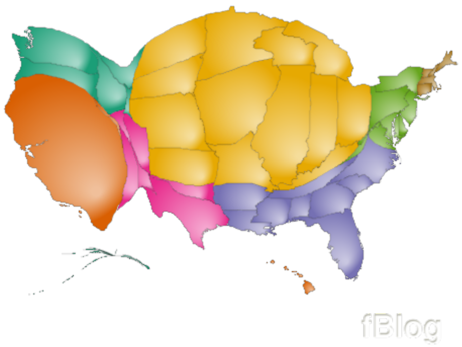

Figure 1b. A cartogram of 2017 crop sales by state in the United States.

Question 4 of 6

_____________ has the highest crop sales in the South East.

A. Arkansas (AR)
B. Florida (FL)
C. North Carolina (NC)
D. Tennessee (TN)

1.5 Task 5

A figure has been generated using the cartogram you uploaded during the previous task. For this task, fill in the blanks correctly. When selecting your answers you may refer directly to the figure or to the fBlog tab open in your browser. When you are finished, click 'Next'.

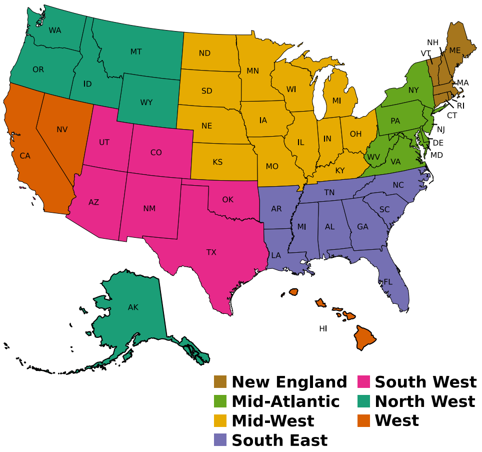

Figure 1a. A land-area map of the United States.

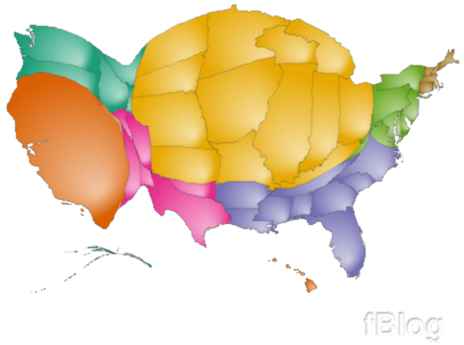

Figure 1b. A cartogram of 2017 crop sales by state in the United States.

Question 5 of 6

The crop sales of Florida (FL) are most similar to those of ____________.

A. South Dakota (SD)
B. Maine (ME)
C. Texas (TX)
D. Minnesota (MN)

1.6 Task 6

A figure has been generated using the cartogram you uploaded during the previous task. For this task, fill in the blanks correctly. When selecting your answers you may refer directly to the figure or to the fBlog tab open in your browser. When you are finished, click 'Next'.

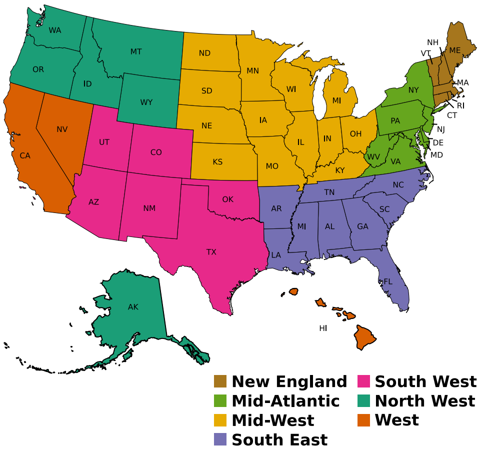

Figure 1a. A land-area map of the United States.

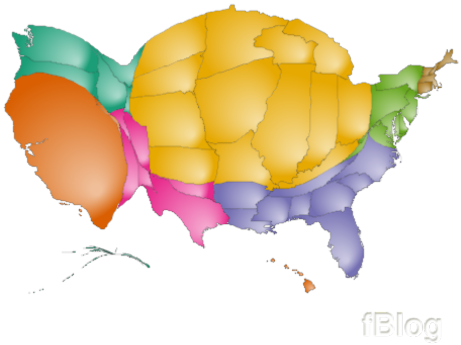

Figure 1b. A cartogram of 2017 crop sales by state in the United States.

Question 6 of 6
The region with the lowest crop sales is _____________.

A. the West
B. New England
C. the North West
D. the Mid Atlantic

2 go-cart.io

2.1 Task 1

A figure has been generated using the cartogram you uploaded during the previous task. For this task, fill in the blanks correctly. When selecting your answers you may refer directly to the figure or to the fBlog tab open in your browser. When you are finished, click 'Next'.

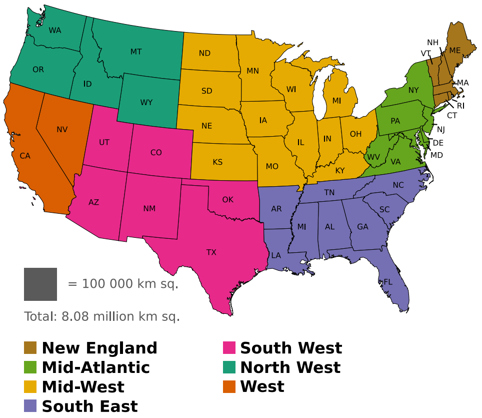

Figure 1a. A land-area map of the United States.

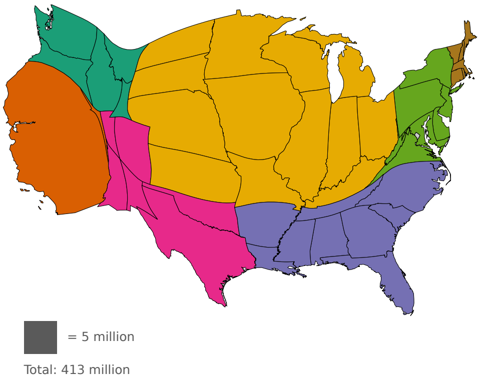

Figure 1b. A cartogram of 2018 agricultural sector output by state in the United States.

Question 1 of 6

______(A)______ has the highest agricultural output, followed by ______(B)______.

	Texas (TX)	California (CA)	Iowa (IA)	Florida (FL)
A				
B				

2.2 Task 2

A figure has been generated using the cartogram you uploaded during the previous task. For this task, fill in the blanks correctly. When selecting your answers you may refer directly to the figure or to the fBlog tab open in your browser. When you are finished, click 'Next'.

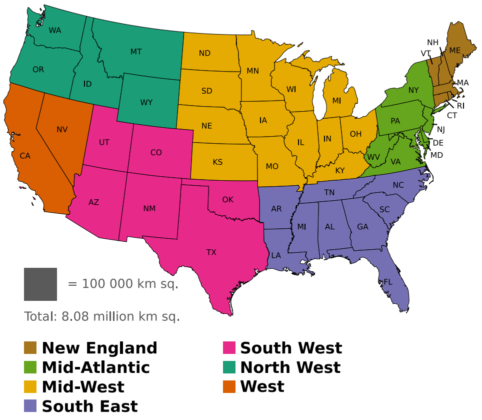

Figure 1a. A land-area map of the United States.

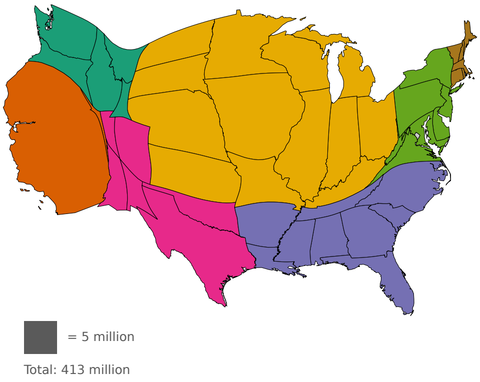

Figure 1b. A cartogram of 2018 agricultural sector output by state in the United States.

Question 2 of 6

America's most productive region is ____________.

A. the Mid-West
B. New England
C. the West
D. the South East

2.3 Task 3

A figure has been generated using the cartogram you uploaded during the previous task. For this task, fill in the blanks correctly. When selecting your answers you may refer directly to the figure or to the fBlog tab open in your browser. When you are finished, click 'Next'.

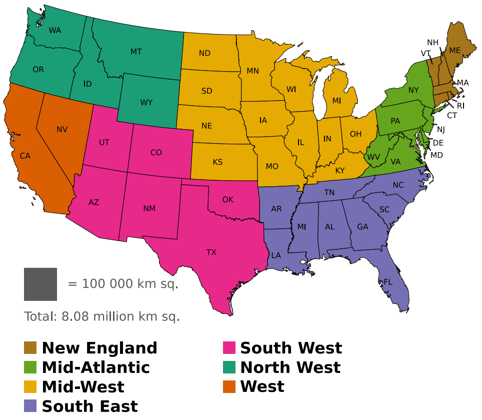

Figure 1a. A land-area map of the United States.

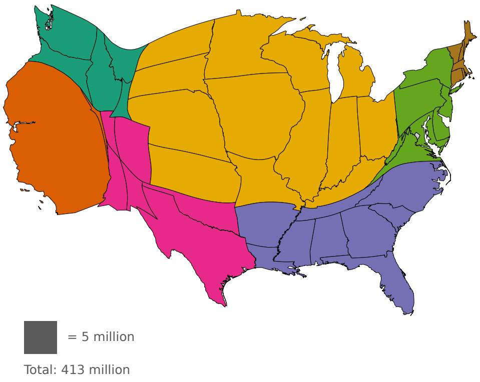

Figure 1b. A cartogram of 2018 agricultural sector output by state in the United States.

Question 3 of 6

The agricultural output of the North West is ____________ that of New England.

A. less than
B. approximately equal to
C. greater than

2.4 Task 4

A figure has been generated using the cartogram you uploaded during the previous task. For this task, fill in the blanks correctly. When selecting your answers you may refer directly to the figure or to the fBlog tab open in your browser. When you are finished, click 'Next'.

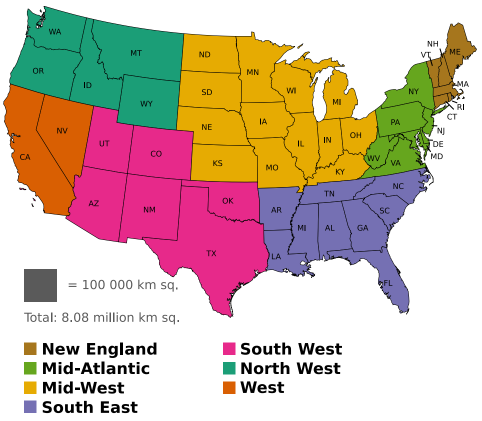

Figure 1a. A land-area map of the United States.

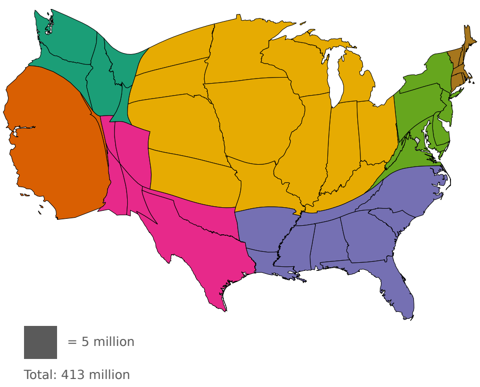

Figure 1b. A cartogram of 2018 agricultural sector output by state in the United States.

Question 4 of 6

____________ is the most productive state in the North West.

A. Idaho (ID)
B. Washington (WA)
C. Oregon (OR)
D. Wyoming (WY)

2.5 Task 5

A figure has been generated using the cartogram you uploaded during the previous task. For this task, fill in the blanks correctly. When selecting your answers you may refer directly to the figure or to the fBlog tab open in your browser. When you are finished, click 'Next'.

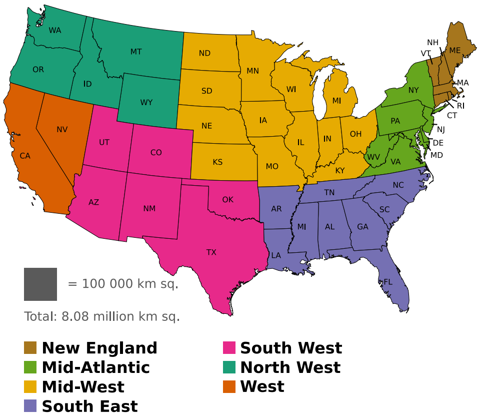

Figure 1a. A land-area map of the United States.

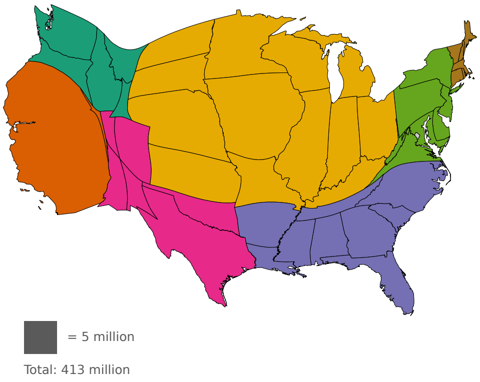

Figure 1b. A cartogram of 2018 agricultural sector output by state in the United States.

Question 5 of 6

The agricultural output of Ohio (OH) is most similar to that of ____________.

A. South Dakota (SD)
B. Maine (ME)
C. Texas (TX)
D. Minnesota (MN)

2.6 Task 6

A figure has been generated using the cartogram you uploaded during the previous task. For this task, fill in the blanks correctly. When selecting your answers you may refer directly to the figure or to the fBlog tab open in your browser. When you are finished, click 'Next'.

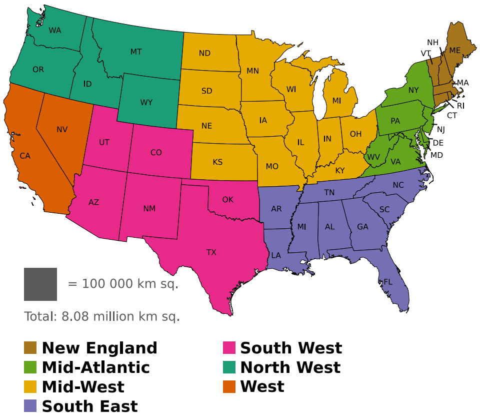

Figure 1a. A land-area map of the United States.

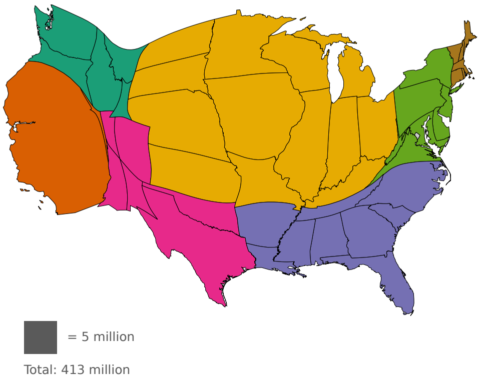

Figure 1b. A cartogram of 2018 agricultural sector output by state in the United States.

Question 6 of 6
The United States' least productive region is ____________.

A. the West
B. New England
C. the North West
D. the Mid Atlantic
