# Supplementary material for: Comparative evaluation of the web-based contiguous cartogram generation tool go-cart.io
Source: PLoS One. 2024 May 8;19(5):e0298192. doi: 10.1371/journal.pone.0298192 (PMC11078394; doi:10.1371/journal.pone.0298192)
Supplement: S4 File — This information was retrieved from https://go-cart.io/cartogram on October 28th, 2023. (PDF) [file pone.0298192.s004.pdf]

List of Maps Pre-Installed in Go-Cart.io

- ASEAN Countries
- Algeria
- Andorra
- Angola
- Arab League
- Argentina
- Australia
- Austria
- Bangladesh
- Belarus
- Belgium
- Bolivia
- Brazil
- Cambodia
- Canada
- Chile
- China (Mainland China and Taiwan)
- Colombia
- Croatia
- Czech Republic
- Denmark
- Dominican Republic
- Estonia
- Ethiopia
- Europe (Eurostat members)
- Finland
- France
- Germany
- Greece
- Guyana
- Hungary
- Iceland
- India
- Indonesia
- Ireland
- Israel
- Italy
- Jamaica
- Japan
- Kazakhstan
- Laos
- Lebanon
- Libya
- Luxembourg
- Malaysia
- Mexico
- Mongolia
- Myanmar
- Nepal
- Netherlands
- New Zealand
- Nigeria
- Pakistan
- Paraguay

- Peru
- Philippines
- Poland
- Portugal
- Qatar
- Romania
- Russia
- San Marino
- Saudi Arabia
- Singapore (by Planning Area)
- Singapore (by Region)
- South Africa
- South Korea
- Spain
- Sri Lanka
- Sudan
- Sweden
- Switzerland
- Thailand
- The Bahamas
- Turkey
- Ukraine
- United Arab Emirates
- United Kingdom
- United States (Conterminous)
- Vietnam
- Washington (U.S. State)
- World
- Yemen
